# The Management of Aortic Coarctation Associated with Hypoplastic Arches and Particular Arch Anatomies: A Literature Review

**DOI:** 10.3390/jpm14070732

**Published:** 2024-07-06

**Authors:** Irina-Maria Margarint, Tammam Youssef, Mircea Robu, Iulian Rotaru, Alexandru Popescu, Olguta Untaru, Cristina Filip, Ovidiu Stiru, Vlad Anton Iliescu, Radu Vladareanu

**Affiliations:** 1 Faculty of Medicine, Carol Davila University of Medicine and Pharmacy, 050474 Bucharest, Romania; irina-maria.margarint@drd.umfcd.ro (I.-M.M.); cristina.filip@umfcd.ro (C.F.); ovidiu.stiru@umfcd.ro (O.S.); vladanton.iliescu@gmail.com (V.A.I.); vladareanu@gmail.com (R.V.); 2 Department of Cardiac Surgery, Emergency Clinical Hospital for Children “Maria Skłodowska Curie”, 077120 Bucharest, Romania; rotaru.iulian7@yahoo.com (I.R.); alexandru.a.g.popescu@gmail.com (A.P.); untaru_olguta@yahoo.com (O.U.)

**Keywords:** aortic coarctation, hypoplastic aortic arch, hypertension, bovine arch, Gothic arch, Romanesque arch

## Abstract

The surgical management of aortic coarctation in newborns needs to ensure postoperative evolution and long-term results as much as possible. Patients with a Gothic arch have a higher rate of postoperative hypertension, while newborns with a bovine arch have higher rates of restenosis and, thus, an additional risk of mortality. Late hypertension, even in anatomically successfully repaired patients, confers a high risk for cardiovascular events. This review of the literature focuses on the management of aortic coarctations associated with hypoplastic arch and particular arch anatomies, focusing on surgical techniques and their outcomes.

## 1. Introduction

Aortic coarctation is a congenital condition that has been well documented and studied. It is typically characterized by a narrowing of the descending aorta, usually located at the level of the aortic isthmus or at the insertion site of the ductus arteriosus, distal to the origin of the left subclavian artery. Aortic coarctation comprises 6–8% of congenital heart disease (CHD) cases, with an incidence of 4/10,000 live births [1]. It has a male preponderance and is frequently associated with other cardiac anomalies such as bicuspid aortic valve, the transposition of the great arteries, ventricular septal defects, patent ductus arteriosus, aortic stenosis, hypoplastic arch, and mitral valve anomalies [2].

Congenital aortic arch anomalies occur due to the maldevelopment and involution of the six pairs of arches that evolve from the paired dorsal aortae [3]. The development of the aorta is complex and occurs during the third week of gestation. The primitive aorta has ventral and dorsal segments. The ventral aorta fuses to form the aortic sac, while the dorsal aorta joins to form the descending aorta. Subsequently, the six pairs of aortic arches develop further, connecting the aortic sac to the dorsal segment. During the fourth week of the embryologic development of the heart, pharyngeal arches receive arteries from the heart. The cardiovascular system results from the development, fusion, or regression of these six pairs of arteries coursing through the pharyngeal arches. Arches I, II, and V regress, arch III forms the carotid system, and arch IV forms the aortic arch on the left and subclavian artery on the right. Arch VI forms the pulmonary arteries and the ductus arteriosus on the left. These developmental milestones and increased blood flow through the aorta at six weeks of gestation are responsible for developing a normal aortic arch. An anomalous development or insufficient increase in blood flow through the aorta can lead to a variety of aortic arch abnormalities that are frequently associated with other types of CHD [4,5].

Aortic coarctation increases blood pressure in the upper limbs, leading to two common presentation patterns [5,6]. The first is neonatal presentation, which is associated with left ventricular dysfunction and shock due to the intolerance of the neonatal myocardium to the sudden increase in afterload that occurs with the closure of the ductus arteriosus. This presentation often occurs one to two weeks after birth [7]. In patients with neonatal coarctation that evolves while the patent ductus arteriosus is closing, the saturation of the lower extremities may be decreased because perfusion to the lower body may be maintained by ductal patency. In the era of lower-extremity pulse oximetry screening in neonates, a newborn may often pass with acceptable saturation, as it is less common for the ductus to contribute significantly unless other left heart structures are hypoplastic. The second presentation occurs in older children and adults. The coarctation of the aorta in this scenario results in hypertension in the upper limbs, leading to early coronary artery disease, aortic aneurysm, and cerebrovascular disease [8]. Coarctation in the neonate may be an emergency when the patient presents with a closed ductus and when it is a critical stenosis [9]. In this situation, it is frequently misdiagnosed with sepsis and needs special attention [10]. After resuscitation of the neonate and stabilization, surgery can be planned within a few days if the patient’s condition allows it [11]. 

The treatment and surgical strategy for aortic coarctation depends on its severity and associated anomalies. The main adverse prognostic factor is late hypertension, with a 20–40% incidence even in anatomically successfully repaired patients. Late hypertension confers a high risk for cardiovascular events [8]. Depending on the associated cardiac defects and the proximal and distal aortic arch size, it can be corrected through a posterolateral left thoracotomy or a median sternotomy. There are many surgical techniques, and they have evolved over the years (end-to-end anastomosis, extended end-to-end anastomosis, end-to-side anastomosis, patch aortoplasty, subclavian flap, reversed subclavian flap), that effectively treat the problem [12].

It is a common occurrence that aortic coarctation is associated with a hypoplastic distal or proximal aortic arch or an aortic arch with a particular anatomy [13]. Because the initial surgical strategy influences the impact on survival and quality of life, the questions investigated are the following: how is the hypoplastic arch/particular anatomy of the arch defined? When the aortic arch is too small for a conventional end-to-end anastomosis, how does the anatomy and geometry of the arch (preoperative or postoperative) influence the risk for hypertension due to modifications of the blood flow through the arch, and does the arch grow to normal values after conventional coarctation repair?

## 2. Methods

We performed a literature review involving pediatric patients with coarctation and a hypoplastic arch who had undergone surgical repair. Articles were searched for on electronic databases—Pubmed, Optechtcs, and Web of Science—between 2001 and 2022, and we selected the articles that addressed the problem of coarctation and a hypoplastic arch or particular arch anatomy. In particular, we reviewed the data available concerning postoperative hypertension, the rate of recoartation, and the influence of exercise capacity on pediatric patients with coarctation repair and particular arch anatomies. Surgical outcomes and different types of coarctation repair were also reviewed. Data were extracted, and quality assessments were undertaken using narrative synthesis. 

The following definitions of a hypoplastic aortic arch were included:

1. An arch with more than a half reduction of its internal diameter.
2. An arch that is smaller than the size of the left carotid artery.
3. An arch that is less than half of the diameter of the ascending or descending aorta or an arch with a z score smaller than −2.

**Figure 1:**
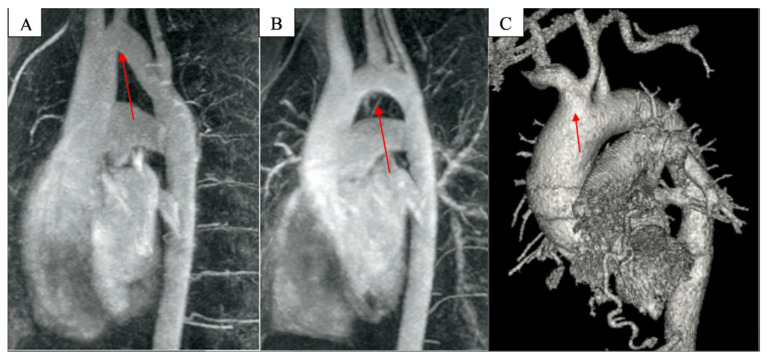
(**A**) Gothic arch, (**B**) Romanesque arch, and (**C**) bovine arch on chest CT angiography.

The particular arch anatomies investigated were the following (Figure 1):

1. Bovine arch: an aortic arch with the innominate artery and left common carotid artery/left common carotid artery both originating from the innominate artery.
2. Based on the geometry in the left anterior oblique position: Gothic, crenel, and Romanesque aortic arches.

**Figure 2:**
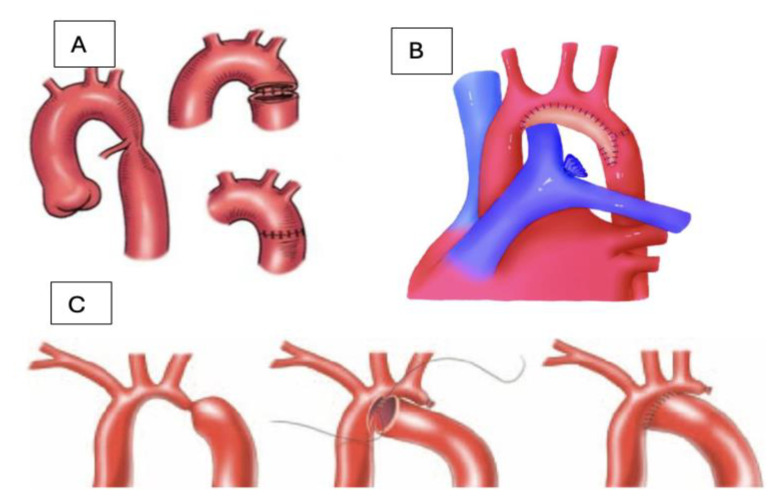
Main surgical options for aortic coarctation repair: (**A**) End-to-end anastomosis; (**B**) patch repair of the aortic arch; (**C**) end-to-side anastomosis.

The main options for repair investigated were the following (Figure 2):

1. End-to-end anastomosis;
2. Extended end-to-end anastomosis;
3. End-to-side anastomosis;
4. The reversed subclavian flap technique;
5. Aortic patch enlargement.

## 3. Review

### 3.1. Definition of Hypoplastic Aortic Arch and Particular Anatomies of the Aortic Arch 

Since there is no clear definition of the hypoplastic arch, multiple authors have tried to investigate this type of arch and the implications for surgery in these patients [14]. In addition to the correct definition of the anatomy, which is of major importance for postoperative results, the definition of the hypoplastic arch in the newborn is of equal importance [15]. According to this, surgical conduct is established. There is no consensus on a single definition regarding the hypoplastic arch [16]. Karl et al. define the hypoplastic arch as an arch smaller than the weight of the baby in kg + 1 [17]. De Leon et al., similar to Tsang, defined patients with proximal arch hypoplasia as having an aortic arch dimension less than their weight in kg plus 1 [18]. 

In addition to the hypoplastic arch, which has smaller dimensions than normal, there are patients with particular arch anatomies that are of normal sizes. These particular arch forms may include the bovine arch and Gothic, crenel, or Romanesque arches [19]. When dealing with a patient with a bovine arch, the first important step is to define and highlight this particular anatomy correctly. Turek et al. defined the bovine aortic arch based on previous definitions, as the arch features a common origin for the innominate artery and left carotid artery or features the left carotid artery originating from the innominate trunk [20]. Phalla et al. reported three types of arches based on aortic arch geometry in the left anterior oblique position: Gothic, crenel, and Romanesque. A Gothic arch is reported to have a triangular form (angulated), as seen in Gothic architecture; a crenel arch has an intermediate rectangular form; and a Romanesque arch has a smooth semicircular form (Figure 1) [21]. 

### 3.2. Impact of Arch Anatomy and Postoperative Hypertension

The authors above report that particular forms of the arch are important in decision-making and surgical strategy. The 49 patients investigated by Turek et al. who had aortic coarctation were treated with extended end-to-end anastomosis through a left posterolateral thoracotomy and followed over 6 years. They evaluated the gradient across the anastomosis and the clampable distance for cases with angiographic images. This distance was compared between patients with normal anatomy and patients with bovine arch anatomy. The clamping distance represents the distance between the left subclavian artery and the maximal proximal clamp location. The results were as follows: the rate of recoarctation in patients with normal arch anatomy was significantly lower than in patients with bovine arch anatomy. In addition, it should be mentioned that all recoarctations happened in infants [20]. The clamping distance for the bovine arch was smaller than in normal anatomy. Because the innominate trunk may be more distantly placed on the arch, the arch can be shorter, so an extended end-to-end anastomosis is more difficult to accomplish. Phalla focused on the risk of postoperative hypertension in patients with particular arch anatomies [21]. They studied 60 patients with aortic coarctation treated either with end-to-end anastomosis or extended end-to-end anastomosis. They studied how the anatomy of the aortic arch affects the appearance of arterial hypertension, even in patients surgically treated for coarctation. They also included in their study 20 healthy volunteers. They analyzed the aortic arch geometry, the aortic stiffness, the extraction of flow wave curves, the aortic pulse wave velocity, the backward systolic flow, and energy dissipation across the arch and the left ventricular mass. Although the patients with a Gothic arch had normal blood pressure at rest, they had a higher pulse pressure. They discovered that there is an earlier and greater volume of backward flow during the cardiac cycle in patients with a Gothic arch. Their pulse wave velocity is greater than in those with a Romanesque arch, and this explains the rigidity of the aorta in the Gothic type of arch. As a link between the pulse wave velocity, aortic stiffness, and hypertension, it also explains the larger left ventricle mass. They concluded that patients with Gothic arches who have undergone a correct coarctation repair have abnormal aortic biomechanics, fluid dynamics, and late hypertension.

Also interested in the relationship between arch shape and hypertension was Bruse et al., who also studied this problem [22]. They investigated 53 patients, 12 to 38 years after coarctation repair, who had no residual obstruction at the level of the isthmus or arch. In total, 80% of the patients were treated by end-to-end anastomosis. They reconstructed the arches in 3D, and from 53 arches they derived a 3D mean anatomic shape and, using deformation vectors, they recreated each analyzed arch. They investigated the relationship between the LVEF (left ventricular ejection fraction), iLVEDV (indexed left ventricular end-diastolic volume), iLVM (indexed left ventricular mass), BP (blood pressure), and arch shape and, after statistical analysis, observed that the derived 3D shape vectors were correlated with LVEF. A Gothic arch with an elevated height-to-width ratio and an elongated ascending and shorter descending aorta are associated with a lower LVEF. Also, these factors were correlated with an increased iLVEDV and higher iLVM. A more rounded shape of arch is associated with a low iLVEDV and low iLVM. A Gothic arch shape with a narrow and short transverse arch was associated with high systolic BP compared to a more rounded type of arch. Although simple coarctation repair is considered a straightforward procedure, there are a great number of patients with hypertension after an effective repair. Thus arises the question of whether arch shape has anything to do with high BP, since arches after coarctation repair look different from those of healthy individuals. It appears that, after visualizing complex arch shapes, the unique arch features after coarctation repair are correlated with a poor LVEF and increased LVEDV and LVM. An aortic arch with an elongated ascending aorta, increased height-to-width ratio, a short transverse arch, and a size mismatch between the isthmus and descending aorta is associated with a poorer LVED. Like other studies, this one demonstrated the association between a particular arch anatomy and hypertension. 

Mandell et al. analyzed the exercise capacity of patients after coarctation repair in terms of their aortic size and shape [23]. They found that the dimension of the descending aorta, the ratio of the ascending to descending aorta diameter, and the flow fraction to the descending aorta at rest are factors associated with exercise capacity. The aorta size mismatch (ascending aorta, descending aorta) was associated with a greater increase in lower body flow fraction between rest and exercise and also with worse exercise capacity. Many studies have focused on aortic arch shape, while this one pays attention to the aortic caliber’s influence on exercise capacity, which seems more important than curvature. A larger ascending aorta and a smaller descending aorta determine a reduced flow fraction to the lower body and correlate strongly with decreased exercise tolerance. 

Phalla et al. took the research further and tried to observe whether the shape of the arch could be distorted during surgery [24]. They studied the effect of aortic shape deformations in patients undergoing coarctation repair on blood pressure. All the patients had their coarctation corrected for 3 months through a left posterolateral thoracotomy either by end-to-end anastomosis or extended end-to-end anastomosis. After investigating patients’ blood pressure at rest and during exercise and making an assessment of the aortic arch through MRI, they categorized their arch anatomy into Gothic, crenel, or normal. Almost half of the patients were hypertensive, with the highest prevalence among those with Gothic arches. To complement their previous study, they then highlighted the association between a Gothic arch and exercise-induced hypertension. 

The shape of the arch is not related to the surgical technique but to the abnormal development of the arch (a much greater growth in height than in width). They also speculate that a short aortic isthmus can predispose the arch to a Gothic anatomy. Bearing this in mind, surgeons must pay attention to the appropriate technique so as not to affect a normal arch’s geometry. A frequently used surgical technique for keeping the normal shape of the arch is the so-called “Norwood type” reported by the Great Ormond Street group [20]. They use a curved patch of pulmonary homograft, thus keeping the normal shape of the arch. 

In addition to Phalla, Cong Li [25] concentrated on the shape of the arch after coarctation repair and, after defining the anatomy of the normal arch, the hypoplastic arch, and coarctation, they investigated 857 patients, of which 121 were under 1 year of age, diagnosed with coarctation or a hypoplastic arch treated by sternotomy and CPB between 2010 and 2020. They aimed to find a proper treatment for patients with proximal or distal hypoplastic arches. The first choice was end-to-side anastomosis (for patients in whom the descending aorta could be fully mobilized and anastomosed to the terminal end of the ascending aorta), but for patients in whom the descending aorta could not be mobilized enough or the anatomy of the aortic arch was particular, they chose a patch repair. At the same operative step, other defects were treated. They used a selection of patches: autologous pericardium (the preferred option), bovine pericardium (if the autologous pericardium was not enough), or pulmonary artery patches harvested from the main pulmonary artery, with the defect created closed using autologous pericardium. Patients with patch plasty had longer aortic cross clamping times than patients treated with end-to-side anastomosis, but their gradients and postoperative course were similar. The mean follow-up time was 1042 days, with 44 patients developing recoarctation, and a higher incidence being observed in the end-to-side group. In 12 patients there was a need for reintervention either by surgery or transcatheter. The authors highlight the importance of tension at the level of anastomosis for avoiding later recoarctation. Regarding end-to-side anastomosis, there are some controversies about its indications. The authors assume that the recoarctation rate is higher with this technique due to a neo-stenosis produced by ascending aortic cannulation and anastomotic ridge formation. The presence of purse-string sutures on the anterior aortic wall may give rise to suture site invagination and anastomosis at the level of the proximal arch, decreasing the length of the transverse arch and enabling the formation of the anastomotic ridge. A technique for suspending the ascending aorta is proposed to avoid late neo-stenosis. By performing a direct anastomosis between the arch and descending aorta, although extensively mobilized, the arch is shortened and the ratio between the width and height of the aortic arch is increased. This new anatomy determines abnormal arch hemodynamics. Although this creates a greater clamping time and bypass time than end-to-side anastomosis, it has similar postoperative outcomes in terms of ICU stay, mechanical support time, and hospital time, which encourage the use of the aortic patch enlargement technique. 

### 3.3. Surgical Management and Outcomes

In search of the most suitable surgical techniques for patients with coarctation and a hypoplastic arch, De Leon et al. described a technique for treating patients with this condition [26]. Patients were treated with aortic arch advancement and ascending sliding arch aortoplasty. All interventions were performed through median sternotomy, bicaval cannulation, and deep hypothermia, with circulatory arrest and selective cerebral perfusion. After cooling the patient, the pump flow is reduced, selective cerebral perfusion begins, and the descending aorta is anastomosed to the proximal arch through an incision on the ascending aorta and arch (arch advancement). In ascending sliding arch aortoplasty, part of the end-to-end anastomosis between the descending aorta and distal arch is performed with antegrade perfusion. After cardioplegia is administered, selective cerebral perfusion is instituted, and the distal ascending aorta is transected, with a tongue of tissue created. This tongue of tissue is advanced on the incision made at the level of the arch and anastomosed. Texas Children’s Hospital has good experience with this technique for patients with coarctation and a proximal hypoplastic arch, with low mortality and low recoarctation rates [27]. 

After defining the anatomy of the coarctation and the hypoplastic arch and choosing the surgical technique, an important issue is whether hypoplastic arches increase in size after the operation or not. Liu et al investigated this matter [28]. After they studied neonates with coarctation and hypoplastic arches treated either by end-to-side anastomosis through sternotomy or through end-to-end anastomosis with conventional thoracotomy, they observed that the proximal arch does not grow to a normal size and patients at late follow-up have z scores smaller than −2. In contrast to the proximal arch, the distal arch has sustained growth, with its maximum in the early postoperative period meaning that, at late follow-up, it has a normal z score. Patients with residual gradients are the ones who had a smaller proximal arch preoperatively. They concluded that patients at risk for hypertension are patients in which the coarctation was repaired through thoracotomy. Because an uncorrected hypoplastic aortic arch leads to late mortality and recoarctation, it should be managed correctly from the start. Although the lack of growth of the proximal arch was clearly shown, the investigators could not find a risk factor for this. 

When treating patients with aortic coarctation, another question arises regarding patients with a hypoplastic arch: should they be operated on conventionally, or is a more extensive surgical intervention, through sternotomy, more beneficial in the long term? 

The studies so far have linked hypertension to a later age at repair, residual obstruction, anomalous arch shapes, and neuro-hormonal mechanisms initiated long before surgery. Rakhra et al. [29] examined 305 patients with small or hypoplastic arches and coarctation, operated on between 1984 and 2004; a quarter of patients were treated through a sternotomy and the rest through thoracotomy. Reobstruction was defined as an arm–leg gradient over 20 mmHg or an echocardiographic gradient over 25 mmHg across the repair site, or reintervention due to an aortic arch obstruction. Also, hypertension was considered if the blood pressure exceeded the 95th percentile and patients were considered prehypertensive if their blood pressure was between the 90th and 95th percentile for their age, sex, and height. After analyzing the data, they found an early mortality of 9% with low weight, complex associated cardiac malformations, and concomitant cardiac procedures being the risk factors. Just eight patients required early reintervention for residual obstructions. Patients treated with end-to-side anastomosis had no reinterventions and their survival at 5, 10, and 20 years was 95%, 94%, and 92%. Freedom from reintervention in this group of patients was 92% at 5 years and 92% at 10 years. Instead, for patients treated with end-to-end anastomosis through thoracotomy, freedom from reintervention was 84% at 5 years and 79% at 10 years. Regarding reobstruction, 92% of patients who received end-to-side anastomosis were free at 10 years compared to just 61% of those who received end-to-end anastomosis. The incidence of hypertension had an increasing prevalence with age and a lower incidence in patients treated with end-to-side anastomosis than in those treated with extensive end-to-end anastomosis. The Melbourne group recommends a more extensive initial surgery because residual arch stenosis is a risk factor for late hypertension and the arch does not grow to normal values. The group utilizes end-to-side anastomosis through median sternotomy as their standard method, as this protects patients from late reintervention. This approach also reduces the risk of late hypertension, a very important mortality factor.

Poirier [30] defines aortic arch hypoplasia as an arch diameter less than 50% of the ascending aorta and/or descending aorta. In an older series of 37 children (ranging from 8 days to 15 years, with a mean age 26 months) in which aortic arch reconstruction was performed between 1982 and 1997 predominantly using patch aortoplasty (in 35 patients), under deep hypothermic circulatory arrest (33 cases)and , employing mostly homografts as the patch material, the authors highlight a high prevalence of previous interventions for aortic coarctation (81%). There were three groups of patients, those with isolated aortic arch hypoplasia, associated intracardiac lesions (VSD, subaortic stenosis, TGA, mitral stenosis, AVSD), or Williams syndrome (pulmonary artery stenosis addressed at the same time), with operative mortality ranging from 0% for the isolated arch intervention to 31% in the associated cardiac lesions group. It is notable that, for the Williams syndrome group, there was 1 death in 4 patients, in total an operative mortality of 13.5%. Neurological complications occurred in four children (10.8%), including seizures, cortical blindness, and choreoathetosis. However, the long-term follow-up (48.96 +/− 37 months) revealed good results, only one late death involving surgery for an intracardiac defect, one balloon angioplasty for distal arch hypoplasia, no additional morbidity, and no gradient larger than 20 mmHg, with no dilatation at the site of the arch plasty.

As we can see, the therapeutic decision is very difficult, with many factors to consider so that patients benefit from the fewest complications, the best short-term results, and the lowest operative risk. Langley et al. investigated this difficult decision when it comes to hypoplastic aortic arches in neonates. They concluded that the first step in decision-making is a clear understanding of the aortic anatomy [28]. During their imagistic investigation, one could easily visualize the ascending aorta, arch anatomy, and branching, as well as calculate the z score values for important structures. Recently, much emphasis has been placed on the anatomy of the supra-aortic vessels and the aortic arch. When echocardiography does not offer a clear image of the anatomy, other types of imagistic investigations should be used. Another important step is defining the hypoplastic arch. It is crucial to effectively treat patients with hypoplastic arches and protect those who do not need a complex intervention. The authors considered patients for surgery when their arch size was smaller than the patient’s weight in kg plus 1, their z score was less than −2, and when the ratio between their arch and descending aorta was less than 50%. From a surgical strategy point of view, there are two options: a single-stage approach or a two-stage approach. The authors opted for a single-stage repair when there were associated cardiac anomalies. Regarding this approach, left posterolateral thoracotomy is preferred for patients with isolated coarctation, while median sternotomy is preferred for a smaller proximal aortic arch.

Is it worth treating these patients with a more extensive operation? When the distal arch is hypoplastic, it can be corrected with an extensive end-to-end anastomosis or an end-to-end anastomosis and subclavian flap without the need to sacrifice the subclavian artery, an effective method published by surgeons in San Donato, Italy [29]. Through a left posterolateral thoracotomy in the third intercostal space, the descending aorta, ductus, aortic arch, and supra-aortic vessels are dissected freely. With separate clamping of the subclavian artery and the aortic arch, proximal to the left carotid artery, and the isthmus, between the left subclavian artery and ductus arteriosus, a longitudinal incision is made on the left subclavian artery, prolonged at the level of the left carotid artery. The vessels’ dorsal walls and ventral walls are anastomosed and then the ductus is ligated, the coarctation resected, and an end-to-end anastomosis is performed. This technique represents a simple and safe option for repairing aortic coarctations with a hypoplastic distal aortic arch [29,31].

Thompson et al. observed the outcomes after this extensive type of repair for aortic coarctations [32]. They included in their study 191 patients with a median age at surgery of 1 month, and the surgical technique was an extended arch repair. Three-quarters of patients had associated intracardiac anomalies. They defined aortic arch hypoplasia as arches smaller than 50% of the ascending aorta’s diameter, and this was observed in half of the patients under 1 year of age. Their surgical approach was an end-to-end anastomosis through left posterolateral thoracotomy and an end-to-side anastomosis through median sternotomy. Early mortality was low and was related to other complex cardiac anomalies. A neurological injury occurred in one patient, and the reintervention rate was low, with the only risk factor being prematurity. Table 1 and Table 2 summarize the main studies investigating surgical techniques and outcomes regarding aortic coarctations and hypoplastic arch/aortic arches with particular anatomies. Table 3 and Figure 2 show the main techniques used in the treatment of aortic coarctations and their advantages and disadvantages. 

## 4. Conclusions

In treating newborns and infants with coarctation of the aorta, surgeons have a great responsibility to ensure a postoperative evolution that is as easy as possible and long-term results that are as good as possible. 

A large number of patients with coarctation of the aorta also have associated hypoplasia of the aortic arch at different levels (proximal, distal, tubular hypoplasia). The definition of a hypoplastic arch varies from arches smaller than the patient’s weight (in mm) plus 1 and arches with a z score less than −2 to arches smaller than 50% of the diameter of the ascending aorta. Also, studies have highlighted multiple types of aortic arches, like bovine arches, Gothic arches, and Romanesque arches, or arches with elongated ascending aorta with a high arch height-to-width ratio, a short proximal arch, and a dilated descending aorta. The size and shape of the aortic arch are important factors that must be evaluated preoperatively and, in cases in which echocardiography cannot provide reliable information regarding the shape and size, other imaging modalities should be used (CT scan, MRI).

After the correct definition of the anatomy and size of the aortic arch and isthmus, the surgeon can move to planning the surgical intervention. This is very much influenced by the information provided by echocardiography or other diagnostic imaging methods. Most centers use a size of the arch smaller than the patient’s weight plus 1 as a factor in their decision to enlarge it. Also, studies discuss and explore cases of arches with dimensions at the limit or at the lower limit but with particular shapes, such as the most common ones: the bovine or Gothic arch. This information should not be neglected in planning the operative technique. Phalla demonstrated that patients with a Gothic arch anatomy have a higher rate of hypertension and backward flow, which are associated with increased rigidity of the aortic wall, and Turek concluded that patients with a bovine arch anatomy have a shorter distance for clamping, which might be the reason for residual stenosis.

Regarding the operative technique, until recently, the preferred technique was an end-to-end anastomosis or extended end-to-end anastomosis. Although there are studies with good results using this type of anastomosis, when considering new research, additional attention is needed when deciding to use this technique. This is suitable for patients with normal aortic arch anatomy and size and with coarctation limited only to the aortic isthmus. A good alternative for patients with normal aortic arch anatomy but coarctation associated with distal aortic arch hypoplasia can be an extended end-to-end anastomosis or the reversed subclavian flap. The surgeon must be aware of the fact that residual stenosis at the level of the anastomosis or stenosis at the aortic arch represents an important risk factor for postoperative hypertension and, thus, an additional risk of mortality. Therefore, each type of arch must be managed accordingly. Rakhra, Liu, and Langley emphasize the fact that there is a growth difference between the proximal and distal arch and a higher incidence of arterial hypertension after conventional coarctation repair, which makes us wonder whether an intervention through a sternotomy approach is more appropriate than through thoracotomy for patients with even a slightly smaller arch and a particular arch anatomy.

The techniques used in the case of patients with coarctation and proximal arch hypoplasia vary depending on the experience of the surgeon, the availability of materials, and the clinic’s preference. Among these techniques, we have mentioned end-to-side anastomosis through sternotomy, aortic arch advancement, and ascending sliding arch aortoplasty or aortic arch plasty with different types of patch materials. The arch can be enlarged through sternotomy, using a CPB and cardiac arrest, with deep hypothermia and selective cerebral perfusion. The results when using these techniques are good in terms of residual stenosis and freedom from reintervention. They lead to better short-term and long-term results than conventional techniques, with a low risk of operative mortality, low risk for reintervention, and better survival. 

Because cardiac surgery is now standardized and the adverse effects of CPB and hypothermic arrest are diminished, more surgeons can opt for an extensive arch repair instead of a conventional end-to-end anastomosis, thus ensuring a normal geometry of the arch and the absence of residual stenoses and avoiding postoperative hypertension.

## Figures and Tables

**Table 1:** Studies on surgical techniques and outcomes regarding aortic coarctations and hypoplastic arch/aortic arches with particular anatomies.

**Author**	**Phalla** [21]	**Phalla** [24]	**Liu** [28]	**Rakhra** [26]	**Giamberti** [29]	**Turek** [20]	**Tsang** [16]
**Year**	2007	2008	2010	2012	2001	2017	2019
**Country**	France, Australia	France	Australia	Australia	Italy	USA	England
**Type of study**	Retrospective	Retrospective	Retrospective	Retrospective	Retrospective	Retrospective	Retrospective
**No. patients**	40	60	20	305	5	49	380
**Mean age**	7.5–32 (mo)	16+/−5 (mo)	9+/−5.6 y	No data	No data	4–5176 days	No data
**Age at surgery (months)**	0.15–120 (mo)	<6 (mo)	8.6+/−5.7 y	5–29 days	4 days	No data	No data
**Arch anatomy**	Excluded patients with abnormal aortic arch shapes, including those with “Gothic” and “crenel” arch geometries	3 aortic arch types: Gothic, crenel, Romanesque	Hypoplastic arch	Hypoplastic arch	Hypoplastic arch	Bovine arch	Not specified
**Surgical approach**	End-to-end anastomosis	End-to-end anastomosis; extensive end-to-end anastomosis	Subclavian flap repair, end-to-end anastomosis, extended end-to-end anastomosis	End-to-side anastomosis, end-to-end anastomosis, extended end-to-end anastomosis, subclavian patch repair	Extended end-to-end anastomosis and modified reverse subclavian flap	Extended end-to-end anastomosis	Norwood type of aortic arch repair
**Outcomes**	No data	Fluid dynamics and aortic biomechanics of the central aorta were markedly abnormal in patients with a Gothic arch; LV mass index was significantly increased in patients with a Gothic arch	1/3 of the patients with a moderately hypoplastic arch treated by conventional coarctation repair kept a small proximal arch (the distal arch tended to increase in size to normal values)	Residual aortic arch obstruction is a risk factor for hypertension after repair; end-to-side repair had a lower reintervention rate than extended end-to-end anastomosis	Successful surgical enlargement of the distal aortic arch while preserving the flow through the left subclavian artery	Increased risk for recurrent arch obstruction due to a reduced clampable distance	Increased preference for sternotomy

**Table 2:** Studies on surgical techniques and outcomes regarding aortic coarctations and hypoplastic arch/aortic arches with particular anatomies.

**Author**	**Bruse** [22]	**Poirier** [30]	**Cong Li** [25]	**Seo** [9]	**Mandell** [23]	**De Leon** [26]
**Year**	2017	1999	2022	2014	2020	2016
**Type study**	Retrospective	Retrospective	Retrospective	Retrospective	Retrospective	Retrospective
**No. patients**	53	37	121	50	15	275
**Mean age**	22.3 ± 5.6 y	26 months	Infants	11 days	26 ± 8.6	
**Age at surgery (mo)**	1–60 (months)	24 ± 26 months	Infants	7–20 days	<1 year–18 years	
**Arch anatomy**	Hypoplastic aortic arch/interrupted aortic arch were excluded from template 3D mean shape	Hypoplastic univentricular physiology, isolated coarctation or recoarctation involving just the isthmus and distal arch were excluded	Hypoplastic, proximal, and distal hypoplasia	Arch obstruction, hypoplastic arch, intracardiac anomalies	Romanesque arch, crenel arch, Gothic arch	Hypoplastic arch
**Surgical approach**	End-to-end anastomosis	Patch aortoplasty from the innominate trunk to the isthmus	End-to-side anastomosis, enlargement of the arch with patch	End-to-side anastomosis, end-to-end anastomosis, modified arch repair technique		Aortic arch advancement, ascending sliding arch aortoplasty
**Outcomes**	Elongated ascending aorta with a high arch height-to-width ratio, short proximal arch and dilated descending aorta associated with lower LVEF, larger iLVEDV, and increased iLVM.	Operative mortality ranging from 0% for the isolated arch intervention to 31% in the associated cardiac lesions group. Long-term follow-up (48.96 +/− 37 months): good results, 1 late death involving surgery for an intracardiac defect, 1 balloon angioplasty for distal arch hypoplasia, no additional morbidity, no gradient larger than 20 mmHg, no dilatation at the site of arch plasty.	No differences between the two techniques regarding the postoperative pressure gradient, intensive care unit stay, in-hospital time, or mechanical support time. Higher recoarctation rate for end-to-side anastomosis.	Reintervention for arch restenosis was performed only in the conventional group. Nine patients with conventional surgery showed abnormal Gothic arch geometry. No Gothic arch geometry in patients treated with the modified technique.	Aorta size mismatch (DAAo/DDAo) predicts exercise capacity in patients with successful coarctation repair due to increased resistance and altered flow distribution .	Recurrence rate: 3% at 6-year follow-up. Perioperative mortality: 3%. Low (4%) rate of incidence for recurrence . Restore normal arch contour using native aortic tissue.

**Table 3:** The main surgical strategies for aortic coarctation repair.

Type of Repair	Advantages	Disadvantages
End-to-end anastomosis	Accessible technique No need for CPB or heart arrest Access through thoracotomy Less invasive	Does not resolve proximal or distal arch hypoplasia Can distort the shape of the arch Applicable in selected simple isolated coarctation cases Can lead to residual obstruction or recoarctation and hypertension
Extended end-to-end anastomosis	Accessible technique Access through thoracotomy Resolves hypoplastic distal arch Less invasive	Does not resolve proximal arch hypoplasia Harder to expose all supra aortic vessels Can distort the arch shape if the anastomosis is under tension Risk for residual arch obstruction and hypertension
End-to-side anastomosis	Resolve proximal and distal arch hypoplasia Lower rate of residual obstruction Lower rate of hypertension	Sternotomy approach Need for CPB and cardiac arrest More technically difficult
Reversed subclavian flap	Effectively resolves distal arch hypoplasia without the need for descending aorta clamping in patients with persistent ductus Can be easily associated with end-to-end anastomosis if, after completion of the anastomosis, the result is not optimal	In patients for whom, after declamping the aorta, the result is not optimal, this can be carried out but the descending aorta must be clamped again May be more technically challenging Resolves only distal arch hypoplasia
Aortic patch enlargement	Effectively relieves the hypoplasia at all levels of the arch Lower risk for residual obstruction Lower risk for hypertension	Access through sternotomy Need for CBP, cardiac arrest and selective cerebral perfusion More technically challenging Dilatation of the patch if not all stenosis are relieved

## Data Availability

Not applicable.
